# Leucine-Rich Repeat (LRR) Domains Containing Intervening Motifs in Plants

**DOI:** 10.3390/biom2020288

**Published:** 2012-06-22

**Authors:** Norio Matsushima, Hiroki Miyashita

**Affiliations:** 1 Division of Biophysics, Center for Medical Education, Sapporo Medical University, Sapporo 060-8556, Japan; 2 Department of Biochemistry, School of Medicine, Sapporo Medical University, Sapporo 060-8556, Japan; Email: h-miyashita@sapmed.ac.jp

**Keywords:** leucine-rich repeats, Non-LRR island, LRR-RLK, LRR-RLP, TMK1, Os10g0479700, TONSOKU/BRUSHY1, MJK13.7, ligand interaction, dimerization

## Abstract

LRRs (leucine rich repeats) are present in over 14,000 proteins. Non-LRR, island regions (IRs) interrupting LRRs are widely distributed. The present article reviews 19 families of LRR proteins having non-LRR IRs (LRR@IR proteins) from various plant species. The LRR@IR proteins are LRR-containing receptor-like kinases (LRR-RLKs), LRR-containing receptor-like proteins (LRR-RLPs), TONSOKU/BRUSHY1, and MJK13.7; the LRR-RLKs are homologs of TMK1/Rhg4, BRI1, PSKR, PSYR1, Arabidopsis At1g74360, and RPK2, while the LRR-RLPs are those of Cf-9/Cf-4, Cf-2/Cf-5, Ve, HcrVf, RPP27, EIX1, clavata 2, fascinated ear2, RLP2, rice Os10g0479700, and putative soybean disease resistance protein. The LRRs are intersected by single, non-LRR IRs; only the RPK2 homologs have two IRs. In most of the LRR-RLKs and LRR-RLPs, the number of repeat units in the preceding LRR block (*N_1_*) is greater than the number of the following block (*N_2_*); *N_1_* » *N_2 _* in which *N_1_* is variable in the homologs of individual families, while *N_2_* is highly conserved. The five families of the LRR-RLKs except for the RPK2 family show *N_1_* = 8 − 18 and *N_2_* = 3 − 5. The nine families of the LRR-RLPs show *N_1_* = 12 − 33 and *N_2_* = 4; while *N_1_* = 6 and *N_2_* = 4 for the rice Os10g0479700 family and the *N_1_* = 4 − 28 and *N_2_* = 4 for the soybean protein family. The rule of *N_1_* » *N_2_* might play a common, significant role in ligand interaction, dimerization, and/or signal transduction of the LRR-RLKs and the LRR-RLPs. The structure and evolution of the LRR domains with non-LRR IRs and their proteins are also discussed.

## 1. Introduction

LRR (leucine rich repeat) regions are present in over 14,000 proteins in the data bases-PFAM, SMART, PROSITE, and InterPro [1,2,3,4]. LRR-containing proteins have been identified in viruses, bacteria, archaea, and eukaryotes. *Arabidopsis thaliana* and *Oryza sativa* subsp. japonica (rice) contain over 700 and 1,400 LRR proteins, respectively [5]. Most LRR proteins are involved in protein-ligand and in protein-protein interactions; these LRR proteins include plant immune response and mammalian innate immune response [6,7,8,9,10]. Most LRR repeating units are 20–30 residues in length. All LRR units can be divided into a HCS (highly conserved segment) and a VS (variable segment). The HCS part consists of an 11 residue stretch, LxxLxLxxNxL, or a 12 residue stretch, LxxLxLxxCxxL, in which “L” is Leu, Ile, Val, or Phe, “N” is Asn, Thr, Ser, or Cys, and “C” is Cys, Ser or Asn [7,11,12,13,14]. Eight classes of LRRs have been characterized by different lengths and consensus sequences of the VS part of the repeats. They are “RI-like”, “CC”, “Bacterial”, “SDS22-like”, “plant specific (PS)”, “Typical”, “TpLRR”, and “IRREKO”. Plant specific LRRs (class: PS-LRR) are 23 to 25 residues long and contain a conserved consensus sequence of the VS part, SGxIPxxLxxLxx, in which “S” is Ser or Thr, “G” is Gly or Ser, “I” is Ile or Leu, and “L” is Leu, Ile, Val, Phe, or Met, and “x” is any amino acid [14]. The structures of polygalacturonase inhibiting protein (PGIP) and brassinosteroid insensitive 1 (BRI1), which have PS-LRRs, are available [15,16,17].

LRR-containing proteins from plants have diverse overall structures and functions. Several classes contain LRR-containing receptor-like kinases (LRR-RLKs) [18,19], LRR-containing receptor-like proteins (LRR-RLPs) [20], nucleotide binding site LRR (NBS-LRR) proteins [21,22] and PGIPs [23,24,25]. They provide an early warning system for the presence of potential pathogens and activate protective immune signaling in plants [26,27,28]. In addition, they act as a signal amplifier in the case of tissue damage, establishing symbiotic relationships and effecting developmental processes.

Evolution of plant, disease resistance (*R*) genes that encode an LRR region has been studied by many researchers [18,22,29,30,31,32,33,34,35,36,37,38,39,40,41,42,43,44,45]. The generations of *R* genes are proposed to be mainly due to gene duplication, genetic recombination, diversifying selection, sequence divergence in the intergenetic region, composition of the transposable elements, gene conversion, and unequal crossover [41,42,43].

Non-LRR, island regions (IRs) interrupting LRRs are widely distributed; they are referred to as “islands” or “loop outs” [46,47]. A large number of plant LRR proteins have non-LRR IRs which are called LRR@IR proteins; they include LRR-RLKs and LRR-RLPs [46,47,48,49,50,51,52,53,54,55,56,57,58,59,60,61]. Some experimental studies on the function of non-LRR IRs within LRR@IR proteins have been performed [62,63,64]. TLRs 7, 8, and 9 out of Toll-like receptors (TLRs) are also LRR@IR proteins [65,66,67]; TLRs initiate an innate immune response [68,69,70,71]. 

A method—LRRpred—identify the repeat number of LRRs and phasing (that is, what segment or residue corresponds to the beginning of a repeating unit) was developed, which incorporates protein secondary structure prediction [65,72]. LRRpred predicts the repeat number and phasing of LRRs to be completely consistent with, or almost so, with those revealed by structure analyses [72]. Furthermore, to identify non-LRR IRs, a method (called LRR@IRpred) utilizing LRRpred was developed and used to find LRR@IR proteins from organisms other than plants [47]. The present article reviews 19 families of plant LRR@IR proteins identified by LRR@IRpred and describes some features of their LRR domains. The structure, function and evolution of the LRR domains as well as the LRR@IR proteins are discussed.

## 2. Structures of Plant LRR Proteins

All of the LRR domains in one protein form a single continuous structure and adopt an arc or horseshoe shape [73]. Three residues at positions 3 to 5 in the HCS, LxxLxLxxNxL or LxxLxLxxCxxL, form a short β-strand. On the inner, concave face there is a stack of the parallel β-strands and on the outer, convex face there are a variety of secondary structures such as α-helix, 3_10_-helix, polyproline II helix, or a tandem arrangement of β-turns, which are connected by two loops. Most of the known LRR structures have caps, which shield the hydrophobic core of the first LRR unit at the *N*-terminus and/or the last unit at the *C*-terminus. In extracellular proteins or extracellular regions, the *N*-terminal and C-terminal caps frequently consist of Cys clusters including two or four Cys residues; the Cys clusters on the *N*- and *C*-terminal sides of the LRR arcs are called LRRNT and LRRCT, respectively [8,9,10].

**Figure 1:**
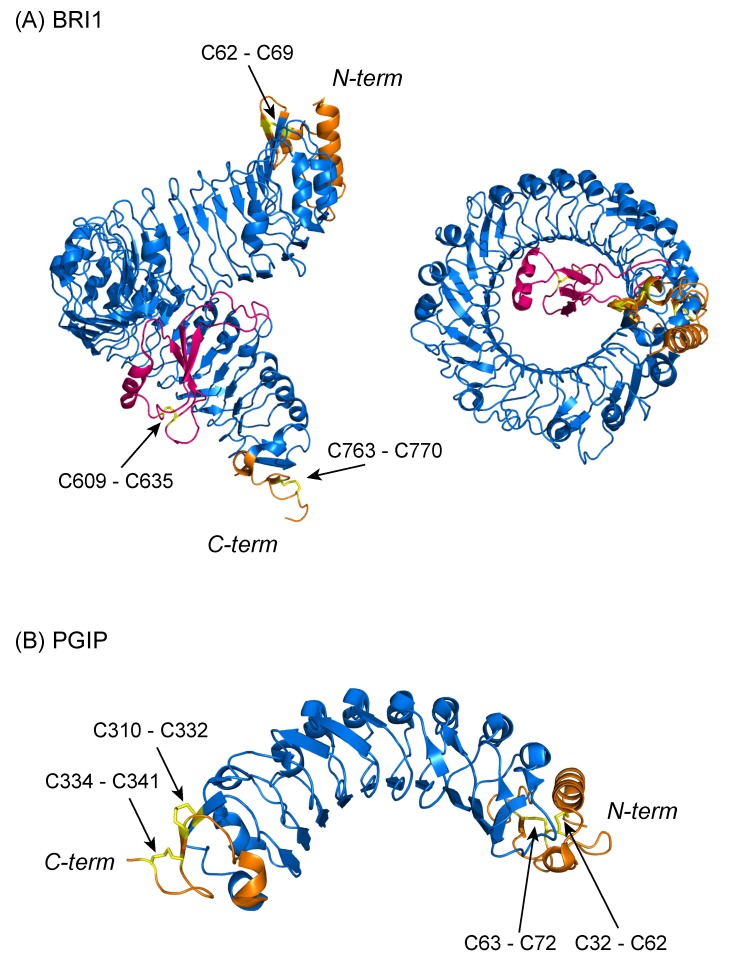
Three-dimensional structures of the PS-LRR domains of BRI1 and PGIP. (**A**) BRI1 [3RGZ]; (**B**) PGIP [1OGQ]. The LRRs are colored blue, the cap structures at the N-terminal and C-terminal side orange, the non-LRR IR in BRI1 pink, and the disulfide bonds yellow. All figures were prepared with PYMOL.

The crystal structures of PS-LRR domains of *Phaseolus vulgaris* PGIP and *A. thaliana* BRI1 (an LRR@IR protein) have been determined [15,16,17]. The structure of the BRI1 LRR domain forms a right-handed superhelix composed of 25 PS-LRRs (Figure 1A) [16,17]; most of these 25 PS-LRRs are 24 residues long. The helix completes one full turn, with a rise of ~70 Å. The concave surface is formed by α- and 3_10_ helices that produce inner and outer diameters of ~30 and ~60 Å, respectively. The consensus sequence LxGx(I/L)P at positions 11 to 16 likely forms a second β-strand, which characterizes the fold of the PS-LRRs. Thus, the structural LRR units may be represented by β-β-3_10_. BRI1 has both an LRRNT with *Cx_6_C* and an LRRCT with *Cx_6_C*; both the LRRNT and LRRCT form two disulfide bonds. The disulfide bonds contribute to the stability of the N-terminal cap structure (*N*-Cap) consisting of one β-strand and two α-helices and the *C*-terminal cap structure (*C*-Cap) consisting of two short helices.

The crystal structures of LRR domains of *A. thaliana* transport inhibitor response 1 (TIR1) and coronatine-insensitive protein 1 (COI1) (that are F-box proteins) are also available [74,75,76]. TIR1 has 18 LRRs of various lengths (from 22 to 35 residues) of which 13 are noncanonical, imperfect LRRs and have long β-strands of 4–6 residues. Most VS parts adopt α-helix. Thus, the structural LRR units may be represented by β-α. The TIR1 LRR domain form a right-handed superhelix of one full turn, which is represented by one closed ring, as well as the BRI1 LRR domain [74,75]. The top surface of the TIR1 superhelix has three long intra-repeat loops (loop-2 in ***LRR2***, loop-12 in ***LRR12*** and loop-14 in ***LRR14***). The loop-2 plays a pivotal role in constructing the auxin- and substrate-binding surface pocket by interacting with the nearby concave surface of the TIR1 LRR structure. The COI1 LRR domain adopts a very similar structure to that of TIR1 [76]. Similarly, three long intra-repeat loops are involved in the bindings of hormone (jasmine) and polypeptide substrates [76].

## 3. Plant LRR@IR Proteins

Plant LRR@IR proteins found through previous research by Matsushima *et al.* [47] and by use of keywords in the references are described. Homologs of an individual protein family from various plant species were collected by the following procedures. First, LRRs in a representative LRR@IR protein of each family were identified by LRR@IRpred; the number of repeat units in the preceding LRR block (*N_1_*), its number in the following block (*N_2_*)*,* and the non-LRR IR sequence of the LRR region were determined. Second, database searches using the amino acid sequences of the non-LRR IR and one LRR unit at the N-terminal and C-terminal IR region were performed by FASTA at the Bioinformatic Center, Institute for Chemical Research, Kyoto University on February 15, 2012. Third, PS-LRR proteins with highly significant similarity (E-value < 10^−10^) were identified and then they were regarded as putative homologs in which the results of amino acid sequence alignments of full lengths and non-LRR IRs, and their domain architecture, were taken account of. Finally, LRRs in the homologs of each family were identified by LRR@IRpred. When a candidate region is not an LRR unit and its length is longer than average length of the repeating unit of LRRs, it was defined as a non-LRR IR. 

The following sequence analyses were also carried out: signal sequence analysis by the program SignalP (http://www.cbs.dtu.dk/services/SignalP/) [77], transmembrane predictions by TMHMM (http://www.cbs.dtu.dk/services/TMHMM/) [78], and the identification of other characteristic regions by SMART (http://smart.embl-heidelberg.de/smart/set_mode.cgi? GENOMIC = 1) [2].

Finally, the 19 families of 344 LRR@IR proteins are described (Supplementary Table S1). The 19 families are grouped into LRR-RLKs, LRR-RLPs, and intracellular proteins. At least one protein in each family has clear experimental evidence for its existence or expression data (such as existence of cDNA(s), RT-PCR or Northern hybridizations) of the existence of a transcript. TMHMM predicts that *A. thaliana* RSYR1 and RPP27 contain a transmembrane region at the N-terminal side (Supplementary Table S1). However, orthology or domain structure was taken account of, and then these two proteins were regarded as LRR-RLKs. SignalP predicts no signal peptide in *A. thaliana* At1g74360 and soybean putative disease resistance protein. Similarly, these proteins were regarded as an LRR-RLK and an LRR-RLP, respectively. 

LRR-RLKs count 165/233/239 proteins from *A. thaliana*, 292/357 proteins from *O. sativa* subst. Japonica (rice) and 440 from *Popula trichocarpa* (poplar) [42,79,80]. LRR-RLPs count 90 LRR-RLPs from rice (*O. sativa*) and 48/56 from *A. thaliana* [42,46]. There are LRR-RLKs and LRR-RLPs having no non-LRR IRs, such as FLS2, Xa21, and TMM [81]. LRR- containing receptor-like cytoplasmic kinases (LRR-RLCKs) that lack an extracellular domain have no non-LRR IRs [79,82].

The present review could not describe all families of LRR@IR proteins in plants because of a limited survey of LRRs having non-LRR, IRs which comes from LRR@IRpred. 

### 3.1. Six Families of LRR-RLKs

**Figure 2:**
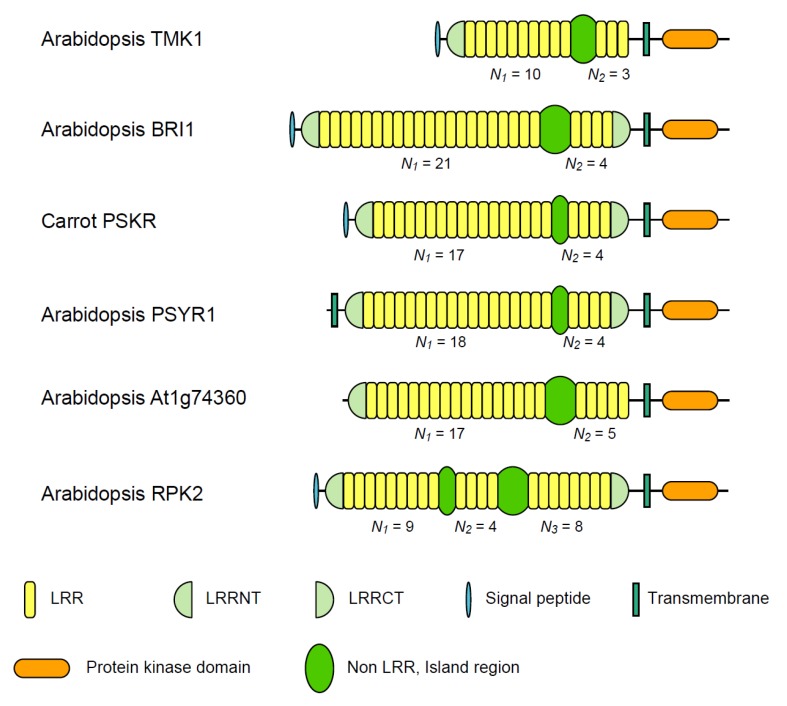
Schematic representation of six LRR-RLKs having LRR domains intersected by non-LRR island regions. *Arabidopsis thaliana* TMK1 [TMK1_ARATH]; *A. thaliana* BRI1 [BRI1_ARATH]; *Daucus carota* PSKR [PSKR_DAUCA]; *A. thaliana* PSYR1 [PSYR1_ARATH]; *A. thaliana* At1g74360 [Y1743_ARATH]; *A. thaliana* RPK2 [RPK2_ARATH].

LRR-RLKs have an extracellular LRR region with an N-terminal signal peptide, a single transmembrane-spanning region, and an intracellular serine-threonine kinase region [18,19],. Transmembrane kinase 1 (TMK1), brassinosteroid insensitive 1 (BRI1), *A. thaliana* At1g74360 protein, phytosulfokine receptor (PSKR), tyrosine-sulfated glycopeptide receptor 1 (PSYR1), and LRR receptor-like serine/threonine-protein kinase RPK2 are members of theLRR-RLKs family. The LRR-RLKs are LRR@IR proteins in which the LRRs are intersected by a single non-LRR IR; only RPK2 has two IRs (Figure 2 and Table 1, and Supplementary Table S1 and Figure S1). 

**Table 1:** Nineteen families of plant LRR proteins having LRR domains intersected by non-LRR island regions. ^a^ “*N*_1_” is the repeat number of LRRs of the first LRR block in the homologs of each family. ^b^ “*N*_2_” is the repeat number of LRRs of the second LRR block in the homologs of each family. ^c^ “*N*_1_/*N*_2_” is average values. ^d^ The LRR domain in Arabidopsis RPK2 contains two non-LRR IRs. The number “13” is the sum of repeat number of LRRs of the first and second LRR blocks. The number “8” is the repeat number of the third LRR block.

	**Nineteen Families of Plant LRR Proteins**	**Species**	**Repeat number of LRRs**			**Lengths of non-LRR**
			*N_1_^a^*	*N_2_^b^*	*N_1_/ N_2_^c^*	Island
(A)	*Six families of LRR-RLKs*					
	ArabidopsisTMK1/Soybean Rhg4	14	8~10	3	3.33	57~61
	Arabidopsis BRI1	24	10~22	4	4.94	67~70
	Carrot PSKR	11	17~18	4	4.36	36~38
	Arabidopsis PSYR1	9	17~18	4	4.47	37~38
	Arabidopsis At1g74360	10	16~17	5	3.40	75~77
	Arabidopsis RPK2	4	13^d^	8^d^	1.63	71~75
(B)	*Eleven families of LRR-RLPs*					
	Tomato Cf-9/Cf-4	8	17~23	4	5.31	41~46
	Tomato Cf-2/Cf-5	3	18~33	4	6.30	37~41
	Tomato Ve	12	28~30	4	7.27	41~49
	Appl HcrVf	1	22~28	4	6.52	39~46
	Arabidopsis RPP27	2	12~26	4	5.78	65~71
	Tomato EIXi	1	27	4	6.75	47~49
	Arabidopsis CLV2	11	18	4	4.50	41~44
	Maize fascinated ear2	4	10~14	4	3.04	41~42
	Arabidopsis AtRLP2	2	18	4	4.50	35~38
	Rice Os10g0469700	6	6	4	1.50	39~40
	Soybean disease resistance protein	5	4~28	4	4.73	41~46
(C)	*Two families of plant intracellular proteins*					
	Arabidopsis TONSOKU	6	10~13	1	12.00	78~131
	Arabidopsis MJK13.7	11	12	8	1.50	59~62

The transcript concentration of *O. sativaTMK1* increase in the rice internode in response to gibberellins [83]. *Nicotiana tabacumTMK1* mRNA accumulation in leaves was stimulated by CaCl_2_, methyl jasmonate, wounding, fungal elicitors, chitins, and chitosan [84]. TMK1 orthologs were identified from 14 plant species and its paralogs are present in 10 species, including *A. thaliana*, *Glycine max,* and *O. sativa* (Figure 2 and Table 1, and Supplementary Table S1 and Figure S1). Also *G.max Rhg4*, which is a soybean cyst nematode resistance gene [85], was identified as a *TMK1* homolog; while *G.max* Rhg1 [C9VZY3] contains 13 PS-LRRs of 24 residues in which only ***LRR6*** is 29 residues long. The TMK1 homologs contain 13 LRRs intercepted by a 56 to 76-residue, non-LRR IR. The number of repeat units in the preceding LRR block (*N_1_*) is greater than the number of the following block (*N_2_*), which means *N_1_* » *N_2_* with *N*_1_ = 10 and *N*_2_ = 3. The non-LRR IRs have a cluster of four Cys residues with the pattern of *Cx_6−7_Cx_29−30_Cx_6−11_C* and a conserved motif of Lx_8_Yx_7*−*8_WxG where “Y” is Tyr or Phe, “W” is Trp, and “G” is Gly; this motif is similar to Yx_8_KG found in many LRR-RLPs [46]. An LRRNT (with *Cx_6_C*) is observed, but not an LRRCT. Putative C-Cap regions are rich in Gly, Ser, and Pro residues. 

BRI1/SR160 is a receptor complex for brassinosteroids that are necessary for plant development, including expression of light- and stress-regulated genes, promotion of cell elongation, normal leaf and chloroplast senescence, and flowering [86,87,88,89,90,91,92]. BRI1 orthologs were identified from 24 species and its paralogs are also present in 10 species. The BRI1/SR160 homologs contain 21–26 LRRs with a single non-LRR IR. The *N_1_* value is relatively variable among species and is 10–22, while *N_2_* = 4; *N_1_* » *N_2_* (Figure 2 and Table 1, and Supplementary Table S1 and Figure S1). *A. thaliana* BRI1 contains 25 LRRs interrupted by a 70-residue IR between ***LRR21*** and ***LRR22.*** The non-LRR IR, together with ***LRR22***, binds brassinosteroids [62]. The non-LRR IRs of the BRI1 homologs are 68–70 residues long and have a cysteine cluster of *Cx_25−26_C* and have a conserved motif of R(I/V/M/L)Y. An LRRNT (with *Cx_6_C*) and an LRRCT (with *Cx_6_C*) were observed; only soybean BR [C6ZRS8] and *Ricinus communis* LRR-RLK [B9T4K2] have LRRNTs with *Cx_26−27_C*. The LRRCT regions are rich in His, Arg, and Lys residues, and thus are basic.

PSKR is a PSK receptor that regulates, in response to PSK binding, a signaling cascade involved in plant cell differentiation, organogenesis, and somatic embryogenesis [55,63,93,94]. PSKR orthologs and paralogs were identified (Figure 2 and Table 1, and Supplementary Table S1 and Figure S1). The PSKR homologs contain LRRs with a 36 to 38-residue, non-LRR IR. *N_1_* = 17 *−* 18 and *N_2_*= 4 (Figure 2 and Table 1, and Supplementary Table S1 and Figure S1). The non-LRR IRs have a conserved motif of (Y/F)x_5*−*12_Yx_5_F. Most LRRCT regions are basic. *Daucus carota* PSKR contains 22 LRRs intersected by a 36-residue IR between ***LRR17*** and ***LRR18***. An LRRNT (with *Cx_33_CCx_6_C*) that is similar to that in PGIP [15] and LRRCT (with *Cx_6_C*) are observed. A 15-residue region within the non-LRR IR is a binding site of PSK [63]. The corresponding regions in the homologs are relatively variable.

*A. thaliana* RSYR1 regulates, in response to tyrosine-sulfated glycopeptide binding, a signaling cascade involved in cellular proliferation and plant growth [95]. The RSYR1 homologs from seven species contain 21–22 LRRs with a 37-residue, non-LRR IR (*N_1_* = 17 − 18 and *N_2_*= 4) (Figure 2 and Table 1, and Supplementary Table S1 and Figure S1). The non-LRR IRs have a conserved motif of Yx_2_LPVFx_4_Nx_4_Qx_2*−*3_QLSxL. The LRRNT (with four, five, or seven Cys residues) and the LRRCT (with *Cx_7_C*) are observed. The LRRCT regions are basic. 

*A. thaliana* At1g74360 is a BRI1-related protein (Figure 2 and Table 1, and Supplementary Table S1 and Figures S1). Putative orthologs and paralogs were identified from 10 species. The At1g74360 family contains 21–22 LRRs with a single IR. The *N_1_* value is relatively conserved among species; *N_1_* = 16 − 17, while not *N_2_*= 4 but *N_2_*= 5. The non-LRR IRs of 76-residue are longer than those in BRI1 and have a cysteine cluster with the pattern of *Cx_25_Cx_16_C*. The IRs are highly conserved among the homologs. 

*A. thaliana* RPK2 is a key regulator of anther development (e.g., lignifications pattern), including tapetum degradation during pollen maturation (e.g., germination capacity) [96,97,98] and contributes to shoot aptical meristerm homeostasis [99,100]. The RPK2 homologs from *Arabidopsis lyrata subsp. Lyrata*, *Populus trichocarpa*, and *R. communis* contain 21–22 LRRs with two non-LRR IRs. The first IR is between ***LRR9*** and ***LRR10***. The second IR is between ***LRR13*** and ***LRR14*** (Figure 2 and Table 1, and Supplementary Table S1 and Figure S1). The second IRs are highly conserved among homologs. There are an LRRNT (with *Cx_6_Cx_25_Cx_16_C*) and an LRRCT (with *Cx_11_C*). The LRRCT region is rich in Ser and Pro residues. Sawa and Tabata [101] have reported the RPK2 homologs from other plant species-*Musa acuminate*, *O.sativa* Japonica Group, *Vitis vinifera*, *Sorghum bicolor*, *Physcomitrella patens*, and *Marchantia polymorpha*.

### 3.2. Eleven Families of LRR-RLPs

**Figure 3:**
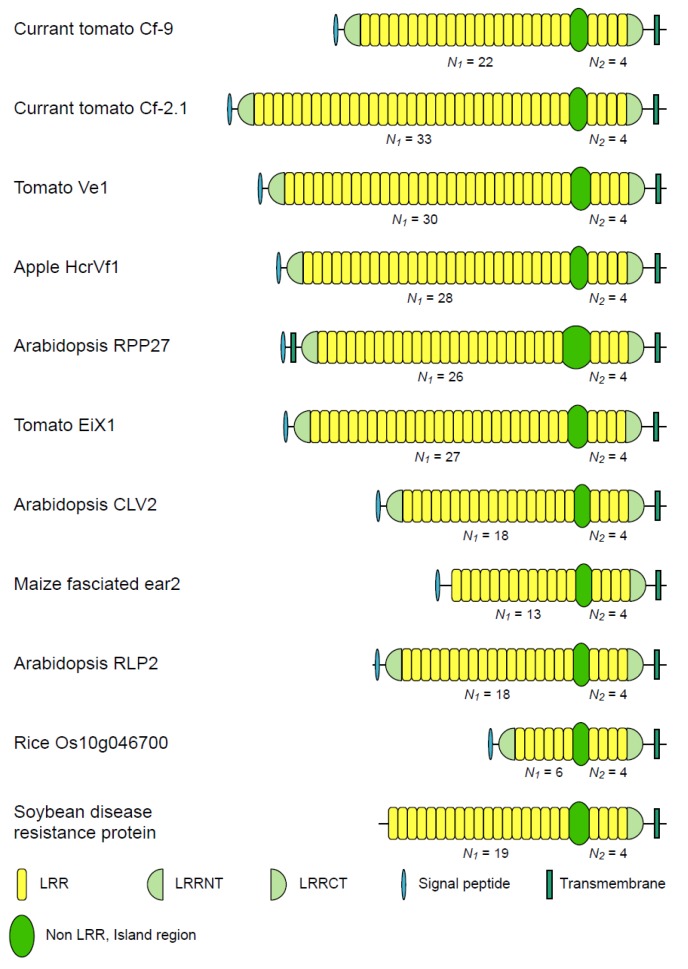
Schematic representation of 11 LRR-RLPs having LRR domains intersected by non-LRR island regions. Currant tomato Cf-9 [Q40235I]; Currant tomato Cf-2.1 [Q41397]; Tomato Ve1 [Q94G61]; Apple HcrVf1 [Q949G9]; *A. thaliana* RPP27 [Q70CT4]; Tomato EIX1 [Q6JN47]; *A. thaliana* CLV2 [Q9SPE9]; Maize fascinated ear2 [Q940E8]; *A. thaliana* RLP2 [RLP2_ARATH]; *Oryza sativa* Os10g0469700 [Q337L7]; Soybean disease resistance protein [C6ZS07].

LRR-RLPs have a short cytoplasmic tail instead of the kinase region in LRR-RLKs (Figure 3) [20]. LRR-RLPs are involved both in resistance of plant–pathogen interactions and development [34,102]. Tomato *Cf* genes confer resistance to the fungal pathogen *Cladosporium fulvum* [43,56,103,104]. Tomato Verticillium wilt disease resistance gens (*Ve1*)and *Ve2*, apple *HcrVf2*, Arabidopsis *RPP27* are involved in resistance to *Verticillium*, *Venturia*, and *Peronospora*, respectively [105,106,107]. Furthermore, the tomato LeEIX initiates defense responses upon elicitation with a fungal ethylene-inducing xylanase (EIX) of non-pathogenic *Trichoderma* from tomato that confer resistance against the fungal pathogen *Cladosporium fulvum* [108,109]. The *clavata2* (*CLV2*) functions in both shoot and root meristems of *Arabidopsis* [58,110,111,112] and also affects autoregulation of nodulation of pea and *Lotus japonicus* [113,114]. *Zea maysfascinated ear2* is involved in meristem development [59]. *A. thaliana* RLP2 is involved in the perception of CLV3 and CLV3-like peptides, that act as extracellular signals regulating meristems maintenance [64]. The LRR-RLPs are all LRR@IR proteins in which the LRRs are intersected by a single non-LRR IR (Figure 3 and Table 1, and Supplementary Table S1 and Figure S1).

Tomato Cf-9/Cf-4 homologs were identified from six species. Elicitor-inducible LRR receptor-like protein (EILP) from *N. tabacum* [115] was identified as ortholog of tomato Cf-9/Cf-4. The number of *N*_1_ is 18 to 22, while *N*_2_ keeps 4, and the non-LRR IRs are 40–44 residues long (Figure 3 and Table 1, and Supplementary Table S1 and Figure S1) and have a conserved motif of MKx_3_Ex_6_Yx_5*−*8_Yx_7_TKG in which hydrophilic residues are conserved. The EILP protein also contains 27 LRRs with *N*_1_ = 23 and *N*_2_ = 4. Most of the homologs have LRRNT consisting of six Cys residues with the pattern of *Cx_24−29_Cx_13−23_CCx_6_Cx_12−13_C*. However, peru 1 and peru 2 have an LRRNT of four Cys’s with the pattern of *Cx_47_CCx_6_C* [116]. The C-terminal side of the LRRCT is rich in Glu and Asp residues and thus is acidic. 

Tomato Cf-2/Cf-5 homologs were identified from two species (*Lycopersicon esculentum,* and *L. pimpinellifolium*). The number of *N_1_* is highly variable; *N*_1_ = 20 − 33, while *N*_2_ keeps 4, and the non-LRR IRs are 37–41 residues long. The IRs are hydrophilic. The variability of *N*_1_ has been reported by other researches in between the paralogs and orthologs [43,46,103,104] (Figure 3 and Table 1, and Supplementary Table S1 and Figure S1). Interestingly, the N-terminal LRRs include tandem repeats of the super-motif of two highly conserved LRRs; for example, LxxLxLxxNxLSGxIPxxIGYLRS and LxxLxLSxNxLNGxIPxxFGxLxN in currant tomato Cf-2.1 [103].

Tomato Ve orthologs and paralogs were identified from twelve species including *Solanum neorickii*, *S. aethiopicum*, *Mentha longifolia*, and *M. spicata* [105,117,118]. The Ve homologs contain 32–34 LRRs intercepted by a 44 to 49-residue, non-LRR IR with *N*_1_ = 28 − 30 and *N*_2_ = 4(Figure 3 and Table 1, and Supplementary Table S1 and Figure S1).The non-LRR IRs have a conserved motif of YYx_8_K(G/R) and are relatively hydrophilic.

Apple *HcrVfs* (**H**omologs of **C**ladosporium fulvum **r**esistance genes of **Vf** region) are scab resistance genes [119,120]. *Mentha longifoliaHcrVfs* are orthologs of tomato *Ve* genes [105,117,118]. The HcrVfs paralogs contain 32–34 LRRs intercepted by a 41 to 46-residue, non-LRR IR with *N*_1_ = 22 − 28 and *N*_2_ = 4 (Figure 3 and Table 1, and Supplementary Table S1 and Figure S1). The non-LRR IRs have a conserved motif of VTKGxExEYx(K/E)ILxFxKxxDLSCNF in which hydrophilic residues are conserved. The C-terminal side of the LRRCT is rich in Gly and Pro residues. 

*A. thaliana* RPP27 homologs were also identified from *A. lyrata.* The LRR@IR proteins contain 16–30 LRRs intercepted by a 61 to 71-residue, non-LRR IR with *N*_1_ = 11 − 26 and *N*_2_ = 4 (Figure 2 and Table 1, and Supplementary Table S1 and Figure S1). The IRs have a conserved motif of FxxKxRYD. The C-terminal side of most LRRCT regions is acidic. 

Tomato LeEIX1 and LeEIX2 contain 31 LRRs intercepted by a 47 to 49-residue, non-LRR IR with *N*_1_ = 27 and *N*_2_ = 4 (Figure 3 and Table 1, and Supplementary Table S1 and Figure S1). The C-terminal side of the LRRCT is acidic.

*A. thaliana* CLV2 homologs were identified from 11 species. The CLV2 homologous proteins contain 22 LRRs intercepted by a 41 to 43-residue, non-LRR IR with *N*_1_ = 18 and *N*_2_ = 4 (Figure 3 and Table 1, and Supplementary Table S1 and Figure S1). The IRs have a conserved motif of LxFxYxL. The C-terminal side of most LRRCT regions is acidic. *A. thaliana* CLV1 is an LRR-RLP but not LRR@IR protein. 

*Z. mays* fascinated ear2 is an ortholog of Arabidopsis CLV2. The homologs were also identified from *O. sativa subsp. Japonica*, and *indica*, and *S. bicolor*. The fascinated ear2 homologous proteins contain 17–18 LRRs intercepted by a 41 to 42-residue, IR with *N*_1_ = 10 − 14 and *N*_2_ = 4 (Figure 3 and Table 1, and Supplementary Table S1 and Figure S1). The IRs and the LRRCT regions are rich in Gly. Both regions may be flexible. 

*A. thaliana* RLP2 contains 23 LRRs that are intercepted by a 44-residue, IR with *N*_1_ = 18 and *N*_2_ = 4 (Figure 3 and Table 1, and Supplementary Table S1 and Figure S1). There are an LRRNT and an LRRCT. The extracellular region including the 23 LRRs is homologous to that in *A. thaliana* PSYR1 [121].

*O. sativa* Os10g0469700 is an LRR@IR protein; the function is unknown (Figure 3 and Table 1, and Supplementary Table S1 and Figure S1). The homologs from four species contain 10 LRRs with a single IR with *N*_1_ = 6 and *N*_2_ = 4. The non-LRR IRs with 39–40 residues is represented by MKxP(K/E)IxSSx_2*−*3_LDGSxYQDRIDIxWKGx_3_FQx_4_L.

A putative disease resistance protein from soybean [C6ZS07] is an LRR@IR protein (Figure 3 and Table 1, and Supplementary Table S1 and Figure S1). The homologs were identified from four species and contain 8–32 LRRs with a single IR with *N*_1_ = 4 − 28 and *N*_2_ = 4. The *N*_1_ number is highly variable in both the paralogs and orthologs. The IRs have a conserved motif of Yx_2_Sx_5_Kx_7_(R/K)I.

### 3.3. Two Families of Plant Intracellular Proteins

*A. thaliana* TONSOKU(TSK)/MGOUN3(MGO3)/BRUSHY1(BRU1), which is localized in the nucleus and is preferentially expressed in the shoot apex than in the leaves and stems, is required for cell arrangement in root and shoot apical meristems and involved in structural and functional stabilization of chromatin [122,123,124]. The TONSOKU protein may represent a link between response to DNA damage and epigenetic gene silencing [125]. 

**Figure 4:**
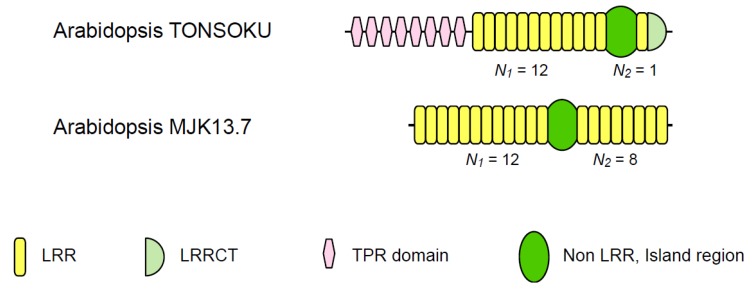
Schematic representation of two plant intracellular LRR@IR proteins having LRR domains intersected by non-LRR island regions. *A. thaliana* MJK13.7 [Q9M7W9]; *A. thaliana* TONSOKU [Q6Q4D0].

Potential homologs of *A. thaliana* TONSOKU have been identified in eight species*.* The UniProKB database describes that *A. thaliana* TONSOKU contains three LRRs and eight TPRs, while the data bases - InterPro, Gene3D, SMART and PROSITE-identify only TPR. LRR@IRpred identifies 14 LRRs with a single IR; *N_1_* = 13, *N_2_* = 1 (Figure 4 and Table 1, and Supplementary Table S1 and Figure S1) [47]. The LRRs are not “plant-specific” motifs but presumably “RI-like” motifs. Thus, the structural LRR units may be represented by β-α instead of β-β-3_10_. The LRR domain is predicted to adopt a typical horseshoe shape seen in ribonuclease inhibitor [126]. The non-LRR IRs are 70–131 residues long and are rich in Ser and Gly. The IRs may be unstructured or flexible. 

*A. thaliana* MJK13.7 is considered to be intracellular protein. The function is unknown. *A. thaliana* MJK13.7 homologs were identified from 11 species. The homologs contain 20 LRRs intersected by a single IR; *N_1_*= 12, *N_2_* = 8 (Figure 4 and Table 1, and Supplementary Table S1 and Figure S1). All of the non-LRR IRs are 60–62 residues long and have conserved Lys residues at five positions. The consensus of the LRRs is LxxLxLxxNxLxxLPxxLxxLxx of 23 residues that are present in many proteins from bacteria to human (data not shown). The LRR motif does not belong to PS-LRR and the structure of the LRR domain is not available. However, the LRR motifs are contained in part of the LRR domains in toll-like receptor 1 (TLR1) and glycoprotein Ibα (GpIbα) of which the crystal structures are available [127,128,129,130]. Four LRRs are**I**KV**L**D**L**HS**N**K**I** K**SIP**KQ**V**VK**L**EA and **L**QE**L**N**V**AS**N**Q**L** K**SVP**DG**I**FDR**L**TS in TLR1, and **L**GT**L**D**L**SH**N**Q**L** QS**LPL**LGQT**L**PA and **L**DT**L**L**L**QENSL YT**IPK**G**F**FGSHL in GpIbαe. The structures revealed that the LRRs may be characterized by extended conformations at the bold sequences [127,128,129,130]. 

Moreover, *A. thaliana* MJK13.7 forms a family with its homologs from insect species, *Strongylocentrotus purpuratus, Nematostella vectensis*, and *Paramecium tetraurelia* and LRRC40 from vertebrates species [47]. The *S. purpuratus* protein has 163 residues containing two repeats of 64 residues each [47]. 

### 3.4. An NBS-LRR Protein

Rice blast resistance gene *Pi-ta* encodes an NBS-LRR protein with 928 residues [44,45]. The Pi-ta protein [Q9AY26] lacks a canonical LRR [44]. The C-terminal region contains highly imperfect LRRs with 10 repeats of various lengths (from 16 to 75 residues) based on the consensus LxxLxxL. The Pi-ta protein appears to be an LRR@IR protein. LRR@IRpred predicts 13 LRRs of 20–54 residues with one non-LRR, IR between ***LRR6* a**nd*** LRR7***(Supplementary Figure S1). The secondary structure prediction prefers α-helix in the VS’s. The Pi-ta LRR domain might adopt a similar structure to those of TIR1 and COI1 [74,75,76]. 

## 4. Features, Structure, Function, and Evolution of the LRR Domains in Plant LRR@IR Proteins

### 4.1. Fundamental Features

Most plant LRR@IR proteins that are LRR-RLKs or LRR-RLPs keep the rule of *N_1_* » *N_2_*;*N_1_* = 10 − 30 and *N*_2_ = 3 − 5 (Table 1). The same rule of *N_1_* » *N_2_*is observed in other LRR@IR proteins of toll receptors and toll-related proteins from insect species, that have one single transmembrane-spanning region and an intracellular Toll IL-receptor (TIR) domain as well as TLRs instead of the kinase region in LRR-RLKs [131]. Most toll receptors and toll-related proteins contain 21–30 LRRs interrupted by a single non-LRR IRs of 81–120 residues with *N_1_* » *N_2_*; *N_1_* = 17 − 24 and *N_2_* = 4 − 6 (data not shown). Fritz-Laylin *et al.* [46] have performed sequence analysis of 90 LRR-RLPs of rice (*O. sativa*) and 56 Arabidopsis (*A. thaliana*). Many LRR-RLPs contain 18–28 LRRs intercepted by a 30 to 80-residue, single IR with *N*_1_ » *N*_2_; *N*_1_ = 14 − 24 and *N*_2_ = 4 [46]. 

The non-LRR IRs in plant LRR@IR proteins may be classified into two groups; one group is non-LRR IRs having cysteine clusters, while the other has no cysteine clusters. The IR cysteine clusters are characterized by *Cx_6−7_Cx_29−30_Cx_7−11_C* in *A. thaliana* TMK1 homologs, *Cx_25_C* in BRI1 homologs, and *Cx_25_Cx_16_C* in At1g74360 homologs. The other non-LRR IRs frequently have a conserved motif of Yx_8_KG which are observed in the homologs of *A. thaliana* TMK1, tomato Cf-9/Cf-4, tomato Cf-2/Cf-5, tomato Ve, *M. longifolia* HCrVf, *A. thaliana* CLV2, and *Z. mays* fascinated ear2, and *O. sativa* Os10g0469700. Non-LRR IRs in many LRR-RLPs from Arbidopsis and rice contain a conserved motif of Yx_8_KG [46]. 

Most of the LRRNTs consist of two, four, or six Cys residues of which the patterns are *Cx_6−7_C*,*Cx_23−34_CCx_6_C*, and *Cx_24−29_Cx_13−23_CCx_6_Cx_12−13_C*. They probably form one, two, and three disulfide bonds, respectively. The LRRCTs consist of two Cys’s with the pattern of *Cx_4−29_C* which probably form one disulfide bond (Supplementary Table S1 and Figure S1). The disulfide bonds should contribute to the structural stabilization of the N-terminal and C-terminal caps.

### 4.2. Possible Structures

The structure of a non-LRR IR is available in *A. thaliana* BRI1 (Figure 1A). The BRI1 LRR domain forms a superhelix with 25 LRRs. The 70-residue, non-LRR, IR in BRI1 between ***LRR21*** and ***LRR22***forms a small domain that folds back into the interior of the superhelix, where it makes extensive polar and hydrophobic interactions with LRRs 13–25 [16,17]. The LRR domain fold is characterized by an anti-parallel β-sheet, which is sandwiched between the LRR core and a 3_10_ helix and stabilized by a disulphide bridge of the Cys cluster with *Cx_25_C* in the non-LRR, IR. Cys clusters are also present in non-LRR, IRs in the homologs of TMK, At1g74360 and TONSOKU. Thus, the non-LRR IRs may adopt similar structures with disulfide bridges. All of the non-LRR IRs would fold back into the interior or exterior of a superhelix of the LRR domains.

### 4.3. Possible Function(s)

The non-LRR IRs of BRI1 and PSKR participate in ligand/protein-protein interactions. The BRI1 non-LRR IR binds brassinosteroids [62]. The insertion of a folded domain into the LRR repeat is probably an adaptation to the challenge of sensing a small steroid ligand [16]. The PSKR non-LRR IR also binds PSK [63]. The non-LRR IRs in TLRs 7, 8, and 9 was also predicted to contribute to nucleic acid-protein interaction [66,132]. 

The non-LRR IRs in plant LRR@IR proteins have frequently conserved motifs that are characterized by hydrophilic residues such as Lys, Arg, Glu and Asp, as noted. Some non-LRR IRs are presumably flexible. The conservation of hydrophilic residues in the IRs is also observed in the respective families of LRRC40, LRRC9, and *C. elegans* LRK-1 which are LRR@IR proteins from organisms including vertebrate other than plants [47]. The IRs might contribute to ligand/protein-protein interactions [47]. Moreover, Afzals *et al*. [133] suggested, based on circular dichroism data, that non-LRR IRs are intrinsically unstructured, providing binding diversity to the domains.

The first LRR block in tomato Cf-9, Cf-4, and Cf-2 recognize fungal avirulence proteins [134,135,136,137,138]. The recognitional specificity of Cf-2 with 37 LRRs lies between leucine-rich repeat ***LRR3***and ***LRR27***, a region that differs from Cf-5 with 31 LRRs by six extra LRR and 78 amino acid substitutions [134]. Although crudely defined, this region of specificity corresponds to those in Cf-4, Cf-9, and Cf-9B responsible for recognition of their cognate ligands [135,136,137,138]. Biochemical studies show that CLV2 is essential for the stability of CLV1, in which CLV1 and CLV2 may form a disulfide-linked heterodimer of 185 kD [58]; CLV1 is an LRR-RLP having no non-LRR IR.

Drosophila Toll and vertebrate TLRs 7, 8, and 9 are LRR@IR proteins [65,66,67] which contain one single transmembrane-spanning region as well as LRR-RLKs and LRK-RLPs from plant. Homo- or heterodimerization are involved in ligand-interactions of vertebrate TLRs [68,69,70,71]. A model for DrosophilaToll activation by ligand Spatzle has been proposed; the first LRR block interacts with Spatzle and the second LRR block forms strong dimer contacts that are prevented by the first block, which in the absence of ligand provides a steric constraint [67,131]. The BRI1 receptor activation involves homodimerization [139]; although Hothorn *et al*., [16] suggested that the superhelical BRI1 LRR domain alone has no tendency to oligomerize, indicating that BRI1 receptor activation may not be mediated by ligand-induced homodimerization of the ectodomain. 

Taken together, non-LRR IRs in plant LRR@IR proteins might participate in ligand/protein-interactions, dimerization or both, although an LRR-RLP, *A. thaliana* CLV2, remains functional without non-LRR IR, while the first and the second LRR blocks are essential for functionality [64]. *N_1_* » *N_2_* brings close proximity of the non-LRR IRs to interact with ligand/protein and a transmembrane region. *N_1_* » *N_2_*might facilitate signaling in the cytoplasm through the ligand/protein- interactions. 

There is a possibility that Cys residues in LRRs are involved in dimerization of LRR@IR proteins. The conserved hydrophobic residues of the PS-LRR consensus sequence of LxxLxLxxNxLSGxIPxxLxxLxx at positions 1, 4, 6, 11, 15, 19, and 22 contribute to the hydrophobic cores in the LRR arcs [8,9]. The conserved hydrophobic residues at positions 1, 19 and 22, and “N” at position 9, are frequently occupied by Cys in the PS-LRRs. Moreover, Cys residues are frequently observed in noncanonical PS-LRRs which, as examples, are longer LRR motifs of 25–30 residues with the consensus of LxxLxLxxNxLSGxIPxxLCxxxxx(x/-)(x/-)(x/-)(x/-)(x/-), in which “-” indicates a possible deletion site. At the present stage it remains unknown whether the Cys residues contribute to the hydrophobic core of the LRR arcs or are exposed to solvent. However, some LRR@IR proteins contain PS-LRRs having Cys at positions 2, 3, or 5 in the HCS part (Supplementary Table S1). The Cys residues are likely to be exposed to solvent in the LRR arc and thus might induce dimerization.

### 4.4. Implications for Evolution

What is the evolutionary origin of non-LRR IRs interrupting LRRs? Previous research provided evidence that a direct duplication of the super motifs containing non-LRR regions naturally leads to the occurrence of non-LRR IRs in LRR@IR proteins, including LRR-containing 17 protein (LRRC17), LRRC32, LRR33, chondroadherin-like protein, trophoblast glycoprotein precursor, and *Leishmania* proteophosphoglycans, not from plants but from other eukaryotes [47]. The non-LRR IRs in plant LRR@IR proteins might originate from such similar events. 

The tomato Cf-2/Cf-5 homologs have PS-LRRs that include tandem repeats of the super-motif of two highly conserved LRRs, as noted [103]. The duplications of the super-motif were suggested to have occurred in the Cf-2/Cf-5 homologs [43]. Super-motifs of LRRs are observed in many LRR proteins. The SLRP subfamily (biglycan, decorin, asporin, lumican, fibromodulin, PRELP, keratocan, osteoadherin, epiphycan, osteoglycin, opticin, and podocan), the TLR7 family (TLR7, TLR8 and TLR9), the FLRT family (FLRT1, FLRT2, and FLRT3), and OMGP [65,140,141] contain tandem repeats of a super-domain of ***STT***, where “***T***” is “typical” LRR and “***S***”is“Bacterial” LRR. Ribonuclease inhibitor also has RI- LRRs consisting of a super-motif of 57 residues that encode two LRRs [142]. The super-repeats as well as Cf-2/Cf-5 have been contributed to the duplication of their super-motifs.

## 5. Evolution of Plant LRR@IR Proteins

A large number of LRR-RLPs resembling the extracellular domains of LRR-RLKs are found in the Arabidopsis genome; although not all RLK subfamilies have corresponding RLPs [121]. Indeed, the present analysis indicates that the extracellular domain in PSYR1 is highly similar to that in RLP2. The same distributions also occur in LRR@IR proteins from other plants, such as *S. bicolor* and *O. sativa* (Supplementary Figure S2). Here four examples are described: Sb10g028170/Sb10g028210 (LRR-RLK/LRR-RLP), and Os06g0691800/Os06g0692700; all the four proteins contain 22 LRRs intersected by a single non-LRR IR of 33 residues with *N_1_* = 18 and *N_2_* = 4. The others are Os07g0597200/Os03g0400850, and OsI_26735/OsI_11946; the LRR-RLKs-Os07g0597200 and OsI_26735 are homologs of Arabidopsis At1g74360. The pair-wise comparisons of the amino acid sequences exceed 50% of the identity in respective pairs. The above observations indicate that the LRR-RLKs and LRR-RLPs evolved from gene duplications and recombination [39].

Two putative uncharacterized proteins from *Z. mays* with 717 residues [B8A2X8] and with 623 residues [B8A383_MAIZE] are paralogs of *Z. mays* TMK1 with 958 residues (Supplementary Figure S2). The 717-residue protein contains 6 LRRs; *N_1_*= 3 and*N_2_* = 3. There are other examples; a hypothetical protein from *Z. mays* with 247 residues [C0PL86] and fasciated ear2 with 613 residues, *O. sativa* Os02g0782800 with 441 residues [Q6K7E5] and BRUSHY1 with 1,332 residues [Q6K7D3]. The occurrence of these proteins is attributed to gene duplication and deletions. 

## 6. Conclusions

Most plant LRR@IR proteins have LRRs intersected a single IR with *N_1_* » *N_2_* in which *N_1_* is variable in their individual homologs, while *N_2_* is highly conserved. For all known LRR-RLPs, *N_1_* = 4. The rule of *N_1_* » *N_2_* plays a common, significant role in ligand-interaction, dimerization, and/or signal transduction of the LRR-RLKs and the LRR-RLPs. All of the LRR domains consisting of PS- LRRs are predicted to form a superhelix and non-LRR IRs in plant LRR@IR proteins fold back into the interior or exterior of the superhelix. The present analyses suggest that some LRR-RLKs and LRR-RLPs evolved from gene duplications and recombination. The present review will stimulate various experimental studies to understand the structure and evolution of the LRR domains with non-LRR IRs and their proteins.

## Supplementary Files

Supplementary File 1 RAR-Document (RAR, 570 KB)
